# Mesencephalic astrocyte-derived neurotrophic factor (MANF) protects against Aβ toxicity via attenuating Aβ-induced endoplasmic reticulum stress

**DOI:** 10.1186/s12974-019-1429-0

**Published:** 2019-02-13

**Authors:** Shengchun Xu, Zemin Di, Yufeng He, Runjie Wang, Yuyang Ma, Rui Sun, Jing Li, Tao Wang, Yujun Shen, Shengyun Fang, Lijie Feng, Yuxian Shen

**Affiliations:** 1 School of Basic Medical Sciences, Anhui Medical University, Hefei, 230032 China; 2 Biopharmaceutical Research Institute, Anhui Medical University, Hefei, 230032 China; 3 Institute of Basic Medical Sciences, Anhui Medical University, Hefei, 230032 China; 4 Center for Biomedical Engineering and Technology, University of Maryland, Baltimore, MD USA

**Keywords:** MANF, β-amyloid, Alzheimer’s disease, ER stress, Apoptosis

## Abstract

**Background:**

Extracellular accumulation of amyloid β-peptide (Aβ) is one of pathological hallmarks of Alzheimer’s disease (AD) and contributes to the neuronal loss. Mesencephalic astrocyte-derived neurotrophic factor (MANF) is an endoplasmic reticulum (ER) stress-inducible neurotrophic factor. Many groups, including ours, have proved that MANF rescues neuronal loss in several neurological disorders, such as Parkinson’s disease and cerebral ischemia. However, whether MANF exerts its protective effect against Aβ neurotoxicity in AD remains unknown.

**Methods:**

In the present study, the characteristic expressions of MANF in Aβ_1–42_-treated neuronal cells as well as in the brains of APP/PS1 transgenic mice were analyzed by immunofluorescence staining, qPCR, and Western blot. The effects of MANF overexpression, MANF knockdown, or recombination human MANF protein (rhMANF) on neuron viability, apoptosis, and the expression of ER stress-related proteins following Aβ_1–42_ exposure were also investigated.

**Results:**

The results showed the increased expressions of MANF, as well as ER stress markers immunoglobulin-binding protein (BiP) and C/EBP homologous protein (CHOP), in the brains of the APP/PS1 transgenic mice and Aβ_1–42_-treated neuronal cells. MANF overexpression or rhMANF treatment partially protected against Aβ_1–42_-induced neuronal cell death, associated with marked decrease of cleaved caspase-3, whereas MANF knockdown with siRNA aggravated Aβ_1–42_ cytotoxicity including caspase-3 activation. Further study demonstrated that the expressions of BiP, ATF6, phosphorylated-IRE1, XBP1s, phosphorylated-eIF2α, ATF4, and CHOP were significantly downregulated by MANF overexpression or rhMANF treatment in neuronal cells following Aβ_1–42_ exposure, whereas knockdown of MANF has the opposite effect.

**Conclusions:**

These findings demonstrate that MANF may exert neuroprotective effects against Aβ-induced neurotoxicity through attenuating ER stress, suggesting that an applicability of MANF as a therapeutic candidate for AD.

**Electronic supplementary material:**

The online version of this article (10.1186/s12974-019-1429-0) contains supplementary material, which is available to authorized users.

## Background

Alzheimer’s disease (AD) is an aging-related, progressive, and clinical incurable neurodegenerative disorder, ultimately leading to dementia. One typical pathological hallmark of AD is senile plaques, composed primarily of amyloid β-peptide (Aβ), which is produced by the proteolytic cleavage of the amyloid precursor protein (APP) by β- and γ-secretase cleavage [1–3]. It is recognized that increased production, oligomerization, and aggregation of Aβ are the crucial factors in the onset of AD [4–6]. Although the precise mechanism is still unknown, it has been proved that Aβ mediates neuronal cytotoxicity which eventually leads to massive neuron loss and degeneration [3, 7].

Recently, multiple studies showed that Aβ aggregation induces endoplasmic reticulum (ER) stress in neurons, which elicits the unfolded protein response (UPR) [8, 9]. Many groups reported that UPR activation is increased in AD patients. ER stress marker immunoglobulin-binding protein (BiP)/GRP78 and UPR-transducer proteins, such as phosphorylated pancreatic ER kinase (p-PERK), eukaryotic initiation factor 2 (eIF2α), inositol-requiring enzyme 1 (IRE1), and activating transcription factor-6 (ATF6), were detected in the hippocampal neurons of AD [10, 11]. If the provoking stress is prolonged, UPR can trigger the cell death program through the activation of C/EBP homologous protein (CHOP), a protein of the C/EBP family of transcriptional regulators, also known as growth arrest- and DNA damage-inducible gene 153 (GADD153) [12]. As neurons are highly susceptible to the toxic Aβ, ER stress-mediated cell death might have an important role in the pathogenesis of AD.

Mesencephalic astrocyte-derived neurotrophic factor (MANF) belongs to the fourth family of neurotrophic factors (NTFs) family, which is an important group of secreted proteins regulating the life and death of neurons during development [13–15]. MANF and its homolog, the cerebral dopamine neurotrophic factor (CDNF), have been identified to protect and rescue midbrain dopaminergic neurons in the rat 6-OHDA model of Parkinson’s disease [15–20]. More importantly, our previous studies show MANF to be a secreted protein, and ER stress upregulates its expression and secretion [13, 14]. It was also found that MANF is widely expressed in mammalian tissues and differently regulated by various ER stress during disease processes [13, 14, 21–26]. Our previous studies show that MANF protects against the neuron death induced by ischemia injury [13, 27–29]. Intracerebral delivery of recombinant human MANF protein (rhMANF) or rAAV-mediated MANF gene also reduces cerebral infarction and neuronal cell apoptosis in stroke animals [29, 30]. Additionally, cytoprotective effects of MANF are not restricted to neurons, also including cardiac myocytes [31], pancreatic insulin-producing β cells [24, 25, 32, 33], retinal cells [26], and so on, suggesting that MANF may have important functions under different pathological conditions.

Owing to the ability of MANF to rescue neuronal loss in several nervous system diseases, we are interested at the profile of MANF induction and potential significance in Aβ pathology. In this study, we showed the increased expressions of MANF and ER stress markers BiP and CHOP in APP/PS1 transgenic mice as well as in the neuronal cells treated with Aβ_1–42_, assessed the protective effect of MANF against Aβ neurotoxicity, and the underlying mechanism was also investigated.

## Methods

### Materials and reagents

Aβ_1–42_ peptide, 3-(4, 5dimemylthiazol-2-y1)-2,5-diphenyltetrazolium bromide (MTT), tunicamycin (TM), and 4′,6-diamidino-2-phenylindole (DAPI) were purchased from Sigma (St. Louis, MO, USA). Neurobasal medium, B27, l-glutamine, Dulbecco’s modified Eagle’s medium (DMEM), and fetal bovine serum (FBS) were purchased from Gibco (Gaithersburg, MD, USA). Recombinant human MANF protein and monoclonal anti-MANF antibody have been described as we previously reported [23, 28, 29].

### Animals

PrP-hAPP/hPS1 double-transgenic mice were from Institute of experimental animals, Chinese academy of Medical Sciences (Beijing, China). The pregnant Sprague-Dawley (SD) rats (grade SPF) were obtained from Anhui Experimental Animal Center (Hefei, China). The animals were kept under standard conditions of temperature (25 ± 2 °C) and lighting (12 h light/dark cycle). The procedure for animal experiments was approved by the Animal Care and the Ethic Committee of Animal Usage of Anhui Medical University.

### Preparation of oligomeric Aβ peptides

Aβ_1–42_ peptide powder was dissolved in phosphate-buffered saline (PBS) to a final concentration of 2 mM. Then, the peptides were snapping frozen in liquid nitrogen. The aliquoted peptide were incubated for 1 week at 37 °C before use, then were dissolved in culture medium to working concentration for treatment. The structure of Aβ peptides were examined under electron microscope. Aβ_1–42_ peptides contained rod and globular shape structure with about 100 nm in diameter.

### Primary neuron cultures

Primary neurons from the cerebral cortex of E17 embryos of pregnant rats were cultured as described previously [29]. Briefly, the embryos of rats were decapitated and the cerebral hemispheres were aseptically removed into Hank’s balanced salt solution (HBSS). After removal of the meninges, the cerebral cortices were cut into small pieces, digested with 0.25% Trypsin-EDTA for 20 min at 37 °C, mechanically dissociated by gentle pipetting with Pasteur pipette, and then centrifuged at 400 g for 5 min. The cell pellet was re-suspended in neurobasal medium supplemented with 2% B27 and 0.5 mM l-glutamine and then cultured on 24-well plates pre-coated with poly-l-lysine at 37 °C and 5% CO_2_ in a humidified atmosphere. Primary cultured neurons at 10–14 days were treated with Aβ peptide or TM following by immunofluorescence staining.

### Cell transfection

Human neuroblastoma SH-SY5Y cells and mouse neuroblastoma N2a cells were obtained from the Institute of Cell Biology, Chinese Academy of Sciences (Shanghai, China). Cells were cultured in DMEM supplemented with 10% FBS and maintained in 5% CO_2_ at 37 °C. MANF-Flag plasmid [13] or the MANF siRNA (5′-GGACCUC A AAGACAGAGAUTT-3′, Shanghai GenePharma Co., Ltd) was transfected into N2a cells with Lipofectamine 2000 (Invitrogen, Carlsbad, CA, USA) according to the manufacturer’s instruction. Twenty-four hours after transfection, N2a cells were treated with Aβ_1–42_ (10 μM) for 24 h or TM (2.5 μg/ml) for 12 h.

### Cell viability assay

Cell viability was evaluated by MTT assay as described previously [23, 34]. In brief, cells were plated into 96-well plates (1 × 10^4^ cells per well) and incubated with Aβ_1–42_ at 37 °C for indicated times. MTT (500 μl, 5 mg/ml) was added to each well and incubated at 37 °C for 4 h, then 100 μl DMSO was added to dissolve the formazan crystals. The absorbance was measured at 570 nm using a microplate reader (Thermo, Varioskan Flash, Finland). Experiments were repeated three times with three replicates each time.

### Flow cytometry with Annexin V/PI double staining

For assessment of apoptosis, cells were stained with Annexin V and propidium iodide (PI) using Annexin V-FITC/PI Apoptosis Detection kit (Bestbio, China) according to the manufacturer’s instructions and subjected to flow cytometry using FACSVerse flow cytometry (BD Biosciences, USA).

### TUNEL assay

Cell apoptosis was measured by the TUNEL kit (Promega, Madison, Wisconsin, USA) according to the manufacturer’s instructions. TUNEL positive cells in five randomly selected fields in at least four independent slices were visualized and counted in a blinded manner under a fluorescent microscope (Olympus, Tokyo, Japan).

### Quantitative real-time polymerase chain reaction

Total RNA was extracted from cells with Trizol reagent (Invitrogen, 15596018) following with reverse transcription to synthesize cDNA. The gene transcripts were quantified by quantitative PCR (qPCR) with SYBR green. The primers are listed as follows: Human *BiP* gene, forward 5′-ACCTGGGTTAGGGTGTGTG-3′ and reverse 5′-TTGCCTGAGT AAAGATGTGG-3′; human *CHOP* gene, forward 5′-GGAGCTGGAAGCCTGG TATGA-3′ and reverse 5′-TCCCTGGTCAGGCGCTCGATTT-3′; human *MANF* gene, forward 5′-TCACATTCTCACCAGCCACT-3′ and reverse 5′-CAGGTCGATCTGC TTGTCATAC-3′; human *GAPDH* gene, forward 5′-CCACTCCTCCACCTTTG-3′ and reverse 5′-CACCACCCTGTTGCTGT-3′. Expressions of gene transcripts were normalized to the levels of GAPDH mRNA. qPCR was carried out by using the ABI7500 instrument (Applied Biosystems, USA).

### Immunohistochemistry

Acetone-fixed brain frozen sections were rehydrated in PBS, and endogenous peroxidase activity was quenched in 0.3% H_2_O_2_ on absolute methanol for 20 min. The sections were incubated with mouse anti-MANF antibody overnight at 4 °C. After washing in PBS, the sections were incubated with the appropriate biotinylated secondary antibodies for 1 h at 37 °C. This was followed by incubation with horseradish peroxidase conjugated streptavidin (HRP-SA) for 15 min at 37 °C. Immunohistochemistry was developed by application of 3,3-diaminobenzidine tetrahydrochloride (DAB) for about 1–3 min. Then the sections were counterstained with hematoxylin, dehydrated in graded ethanol, cleared in xylene, and then observed under light microscopy.

### Immunofluorescent staining

Cells were fixed with paraformaldehyde, permeabilized/blocked in PBS containing 0.5% Triton X-100 and 5% BSA. The cells were incubated with following primary antibodies: rabbit anti-BiP antibody (1:500, proteintech, 11587-1-ap), rabbit anti-CHOP antibody (1:400, proteintech, 15204-1-AP), or mouse anti-MANF antibody overnight at 4 °C, followed by Alexa Fluor 488-conjugated or 568-conjugated IgG (1:500, Invitrogen, A11029, A11036) at 37 °C for 1 h; the nuclei of cells were stained with DAPI (5 mg/ml). Images were taken under fluorescent microscopy (Olympus, Tokyo, Japan) with constant parameters of acquisition. Immunofluorescent staining of brain slice was performed as described previously [29]. The following primary antibodies were used: rabbit anti-NeuN antibody (1:100, Abcam, ab177487), rabbit anti-BiP antibody (1:500, proteintech, 11587-1-ap), rabbit anti-CHOP antibody (1:400, proteintech, 15204-1-AP), or mouse anti-MANF antibody.

### Western blot

The cell lysate was prepared for SDS-PAGE as described previously [23, 34]. The proteins were transferred to PVDF membranes and blocked in 5% nonfat milk at room temperature for 1 h, then incubated at 4 °C overnight with the following primary antibodies: rabbit anti-MANF antibody (1: 1000, Abcam, ab67271), rabbit anti-BiP antibody (1:1000, proteintech, 11587-1-ap), rabbit anti-CHOP antibody (1:1000, proteintech, 15204-1-AP), rabbit anti-phospho-eIF2α antibody (1:1000, CST, 3398 s), rabbit anti-phospho-IRE1 antibody (1:1000, Bioss, bs-4308R), rabbit anti-XBP1s antibody (1:1000, Biolegend, 619502), mouse anti-ATF4 antibody (1:1000, CST, 11815 s), rabbit anti-ATF6 antibody (1:1000, Proteintech, 24169-1-AP), rabbit anti-cleaved-caspase 3 antibody (1: 1000, CST, 9664S), mouse anti-α-tubulin (1: 1000, sigma, t6199), and rabbit-anti-GAPDH (1: 1000, Elabscience, E-AB-20059). After washing several times in TBST, membranes were incubated with horseradish peroxidase-conjugated secondary IgG for 1 h. Blots were developed using the enhanced chemiluminescence kit (Amersham Biosciences, NJ, USA). The densitometric analysis was performed using ImageJ software.

### Statistical analysis

The data were expressed as mean ± SD from at least three independent experiments and analyzed using a one-way analysis of variance (ANOVA) with Tukey’s post-hoc tests. A value of *p* less than 0.05 was considered to be statistically significant.

## Results

### Aβ_1–42_ decreased cell viability and induced apoptosis in neuronal cells

**Fig. 1:**
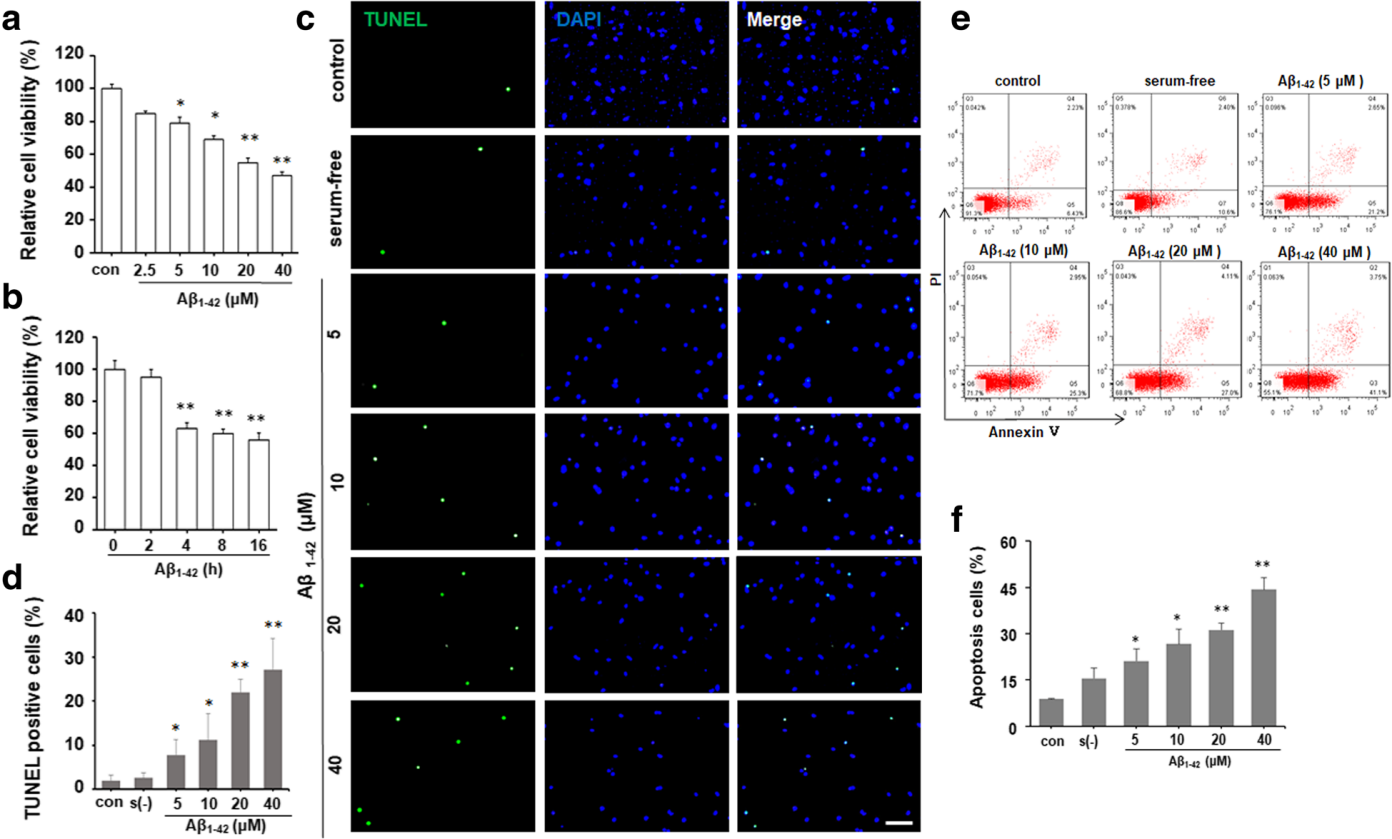
Aβ_1–42_ decreases cell viability and induced cell apoptosis in human neuroblastoma SH-SY5Y cells. MTT assay showed a significant decrease in the viability of SH-SY5Y cells after Aβ_1–42_ exposure in a dose (**a**) and time-dependent (**b**) manner. *P < 0.05, **P < 0.01 compared with control. **c** SH-SY5Y cells were incubated with DMEM containing 10% FBS, serum-free DMEM, or DMEM containing different concentrations of Aβ_1–42_ (5, 10, 20, and 40 μM) for 24 h, respectively. The apoptotic cells were detected by TUNEL staining (green). DAPI was used to determine the number of gross nuclei. Scale bar = 50 μm. **d** Quantitative analysis of the number of TUNEL-positive cells in **c**. *P < 0.05, **P < 0.01, compared with serum-free group. **e** FACS analysis using Annexin V/PI staining showed the increased cell apoptosis in SH-SY5Y cells treated with different concentrations of Aβ_1–42_ for 24 h compared with serum-free group. **f** The quantitative analysis of the number of apoptotic cells in e. *P < 0.05, **P < 0.01, compared with serum-free group. All the quantitative data were presented as mean ± SD of at least three independent experiments. *s*(−) serum-free, *con* control

**Fig. 2:**
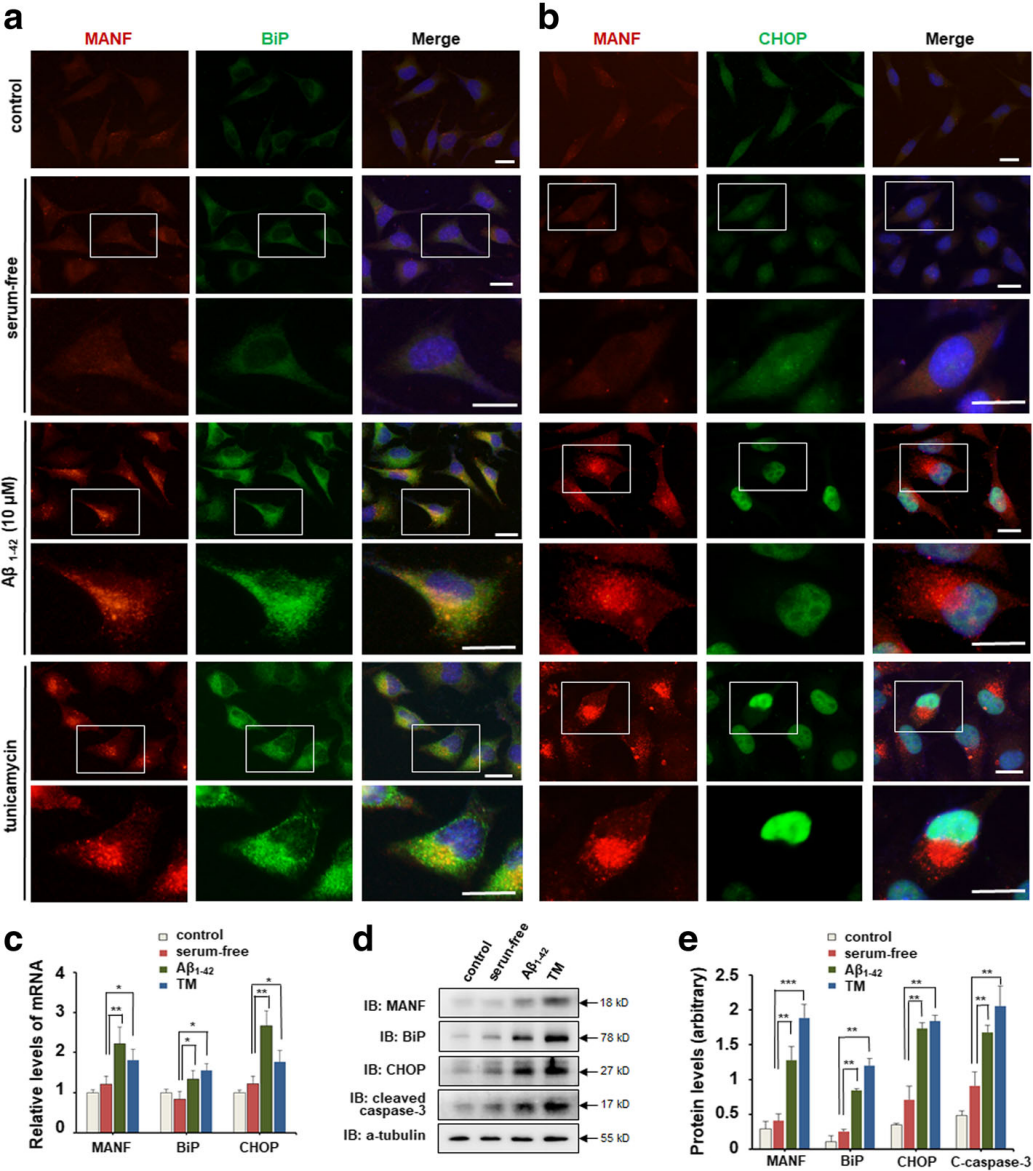
Aβ_1–42_ induces ER stress and up-regulated MANF expression in SH-SY5Y cells. **a** Representative images of MANF (red) and BiP (green) immunofluorescence labeling of SH-SY5Y cells incubated with DMEM containing 10% FBS, serum-free DMEM, and DMEM containing Aβ_1–42_ (10 μM) for 24 h, or DMEM containing tunicamycin (TM) (2.5 μg/ml) for 12 h, respectively. The nuclei were stained with DAPI (blue). The enlarged images show the expression and cellular distribution of MANF and BiP, respectively. Scale bar = 10 μm. **b** Representative images of MANF (red) and CHOP (green) immunofluorescence labeling of SH-SY5Y cells incubated with 10% FBS, serum-free DMEM, and DMEM containing Aβ_1–42_ (10 μM) for 24 h, or DMEM containing TM (2.5 μg/ml) for 12 h, respectively. The enlarged images show the expression and cellular distribution of MANF and CHOP, respectively. Scale bar = 10 μm. **c** Quantitation of *MANF*, *BiP*, and *CHOP* mRNA levels in SH-SY5Y cells treated with DMEM containing 10% FBS, serum-free DMEM, DMEM containing Aβ_1–42_ (10 μM) for 24 h, or DMEM containing TM (2.5 μg/ml) for 12 h, respectively. *GAPDH* was used as control. **d** The protein levels of MANF, BiP, CHOP, and cleaved caspase-3 in SH-SY5Y cells treated as indicated. α-tubulin was used as a loading control. **e** Quantitation of protein levels normalized to the α-tubulin by densitometry in d. All the quantitative data were presented as mean ± SD of at least three independent experiments.*P < 0.05, **P < 0.01. *C*-*caspase*-*3* cleaved caspase-3, *TM* tunicamycin

SH-SY5Y cells were treated with different concentrations of Aβ_1–42_ (2.5, 5, 10, 20, and 40 μM) for 24 h or 10 μM Aβ_1–42_ for different times (2, 4, 8, and 16 h). MTT results revealed that Aβ_1–42_ inhibited cell viability in dose- and time-dependence manner (Fig. 1a, b). TUNEL staining was used to distinguished apoptotic cells from viable cells. Aβ_1–42_ significantly increased the percentage of TUNEL positive cells especially at 20 and 40 μM (Fig. 1c, d). Flow cytometry with Annexin V/PI double staining also confirmed that treatment with Aβ_1–42_ dose-dependently increased the number of apoptotic cells (Fig. 1e, f). Consistently, the level of cleaved caspase-3 protein was dramatically induced by Aβ_1–42_ or ER stress inducer TM (2.5 μg/ml) treatment (Fig. 2d, e). These results suggest that Aβ_1–42_ decreased neuronal cell viability and induced cell apoptosis.

### Aβ_1–42_ induces ER stress and upregulates MANF expression in neuronal cells

To clarify whether Aβ_1–42_ treatment leads to ER stress in neuronal cells, the levels of BiP, an ER chaperone which is known to be induced by ER stress [35], was determined by using immunofluorescent staining and WB in SY5Y cells. Different from the weak expression of BiP in the control or serum-free group, significant increased fluorescence signal of BiP was detected in Aβ_1–42_-treated cells as well as in TM-treated cells (Fig. 2a, enlarged panel). CHOP is a stress-inducible nuclear protein [36], and was usually used as a marker of ER stress-induced cell apoptosis [37]. Immunofluorescent staining revealed a robust increase in CHOP immunoreactivity with distinct nuclear localization in Aβ_1–42_-treated cells (Fig. 2b, enlarged panel). Similar results were observed in SY5Y cells treated with TM (Fig. 2b, enlarged panel). Compared with the serum-free group, mRNA and protein levels of BiP and CHOP were dramatically elevated in Aβ_1–42_-treated SY5Y cells, as well as in TM-treated cells (Fig. 2c–e). Increased immunoreactivity of BiP and CHOP was also shown in primary cultured neurons treated with Aβ (Additional file 1: Figure S1). These results suggest that Aβ_1–42_ induces ER stress in neuronal cells.

Earlier study shows that MANF is an ER stress-sensitive protein [13], so we are wondering if Aβ exposure could upregulate MANF expression. The small amount of MANF was found in control cells and exhibits diffuse distribution in cytoplasm, whereas immunoreactivity of MANF was obviously increased in cells treated with Aβ_1–42_ as well as in TM-treated cells (Fig. 2a, b), which is consistent with our data in the primarily cultured neurons (Additional file 1: Figure S1). Furthermore, both Aβ- and TM-induced expression of MANF were found to be perinuclear distribution (Fig. 2a, and b, enlarged panel). We also found that the protein and mRNA levels of MANF in Aβ_1–42_-treated cells were much higher than that in serum-free group (Fig. 2c–e). These results suggest that Aβ exposure upregulates MANF expression in neurons.

### ER stress and MANF expression in the brains of the APP/PS1 double transgenic mice

**Fig. 3:**
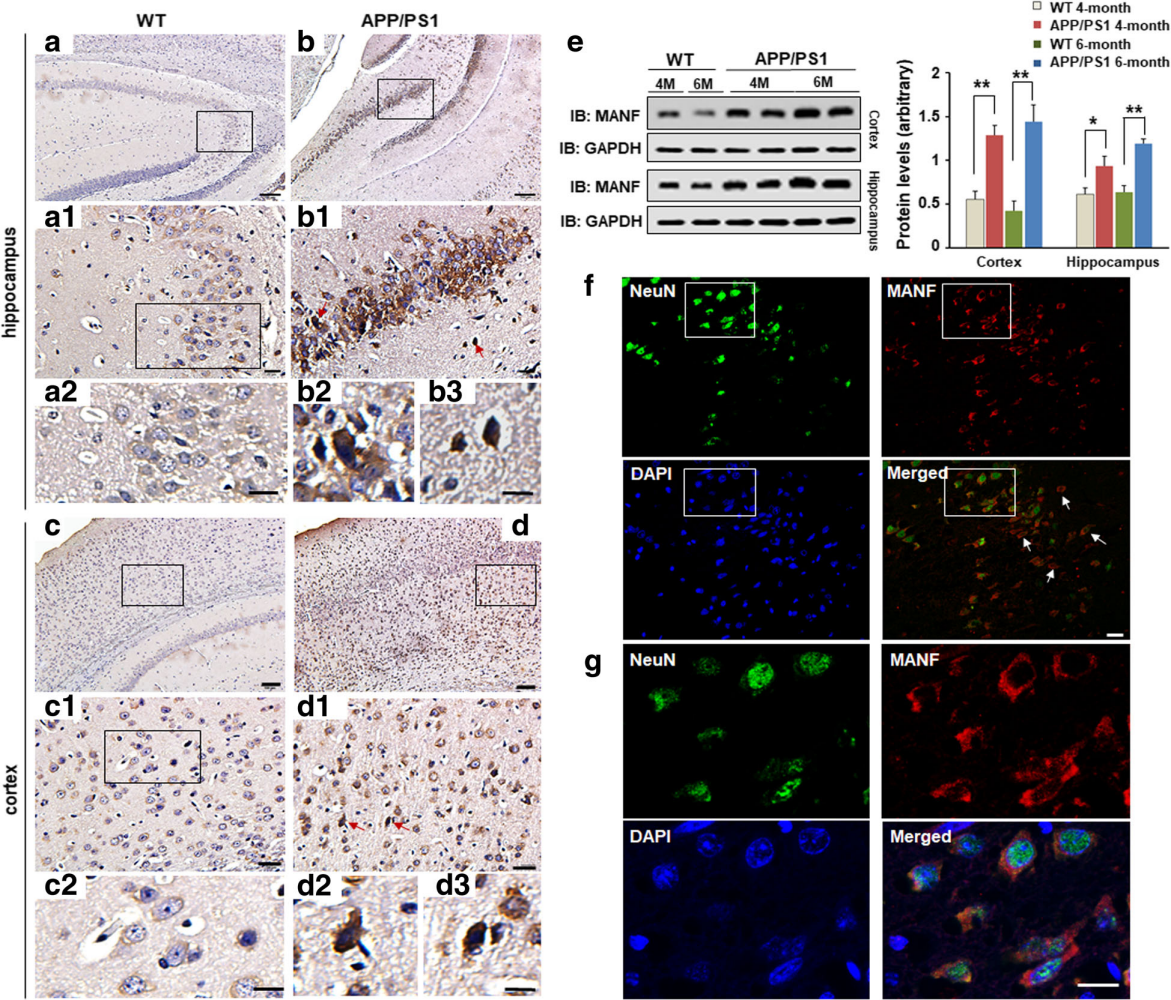
MANF expression in the brains of the APP/PS1 transgenic mice. DAB staining of MANF in hippocampus (a, b) and cortex (c, d) of WT mice (a, c) and APP/PS1 mice (b, d) aged 6 months. Noted the upregulated MANF expression in the brains of APP/PS1 mice (b1, d1) compared to WT mice (a1, c1). Panels a2 and c2 are magnified images in a1 and c1, respectively. Panels b2, b3, and panels d2, d3 are enlarged images in b1 and d1 (red arrows indicated) respectively, to show cellular distribution of MANF in hippocampus and cortex. Scale bar = 100 μm (a–d) or 20 μm (a1, a2, b1, b2, b3, c1, c2, d1, d2, and d3). (e) The levels of MANF in the hippocampus and cortex of 4- and 6-month-old male APP/PS1 mice and age- and sex-matched wild-type (WT) mice, respectively. Quantitative analysis of MANF levels is shown in the right panel. Data are presented as mean ± SD, four mice were used for each group. *P < 0.05, **P < 0.01. (f) Representative images of MANF (red) and NeuN (green) immunofluorescence labeling in the cerebral cortex of the APP/PS1 transgenic mice. The nuclei were stained with DAPI (blue). (g) The magnified images in f to show the colocalization of MANF and NeuN. Scale bar = 10 μm. *M* month, *WT* wild-type

To further confirm the relationship between Aβ pathology and increased MANF, we observed the expression characteristic of MANF in the brains of the wild-type or APP/PS1 double transgenic mice which exhibit remarkable β-amyloid deposits in the brain (Additional file 2: Figure S2). It was found that MANF was detectable in many brain regions of age-match WT mice, including hippocampus (Fig. 3(a, a1, a2)) and cortex (Fig. 3(c, c1, c2)), but the staining is weak. Conversely, the immunoreactivity of MANF was significantly increased in the hippocampus (Fig. 3(b, b1, b2, b3)) and cortex (Fig. 3(d, d1, d2, d3)) of 6-month-old APP/PS1 mice. To further investigate the subpopulations of MANF-positive cells, we performed the double immunofluorescent staining with antibodies against MANF and NeuN, a neuron-specific nuclear protein. We showed that MANF is mainly expressed in neurons, although some NeuN-negative cells also presented MANF-positive immunoreactions. (Fig. 3(f, g)), Western blot shown in Fig. 3(e) also confirmed that the level of MANF is significantly increased in the brains of APP/PS1 mice compared with that of age- and sex-matched WT mice, especially in 6-month-old APP/PS1 mice. Furthermore, BiP- or CHOP-positive cells were largely dispersed in the cortex and hippocampus of the APP/PS1 mice compared with that of WT mice (Additional file 3: Figure S3). Above results suggest that ER stress and MANF expression were induced in the brains of APP/PS1 transgenic mice.

### MANF protected neuronal cells against Aβ toxicity

**Fig. 4:**
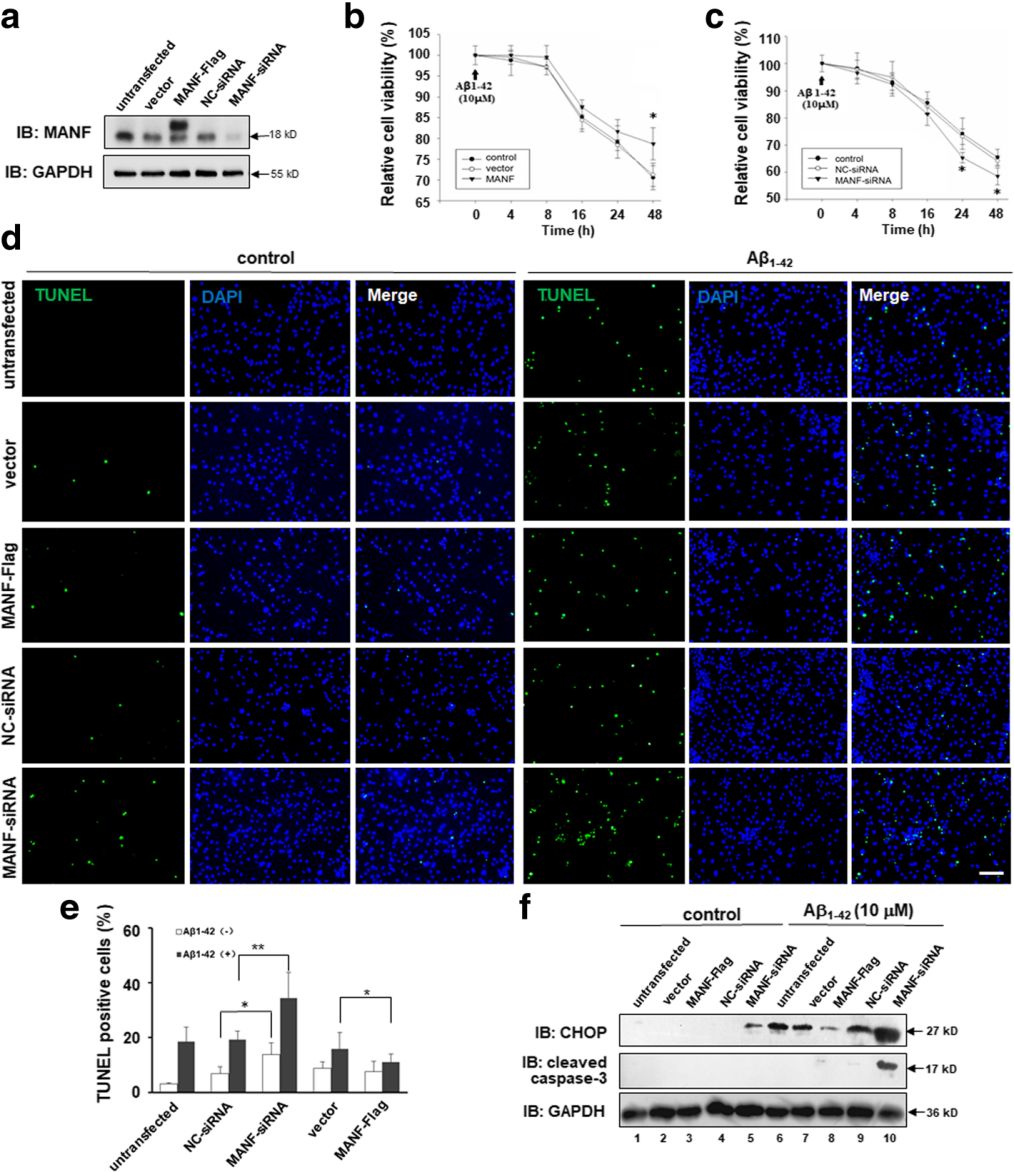
MANF protects against Aβ_1–42_-induced cell toxicity. **a** MANF level in N2a cells transfected with MANF-Flag plasmid or MANF-siRNA. **b** MANF overexpression increases the cell viability in Aβ_1–42_-treated cells compared with that of pcDNA3.1-vector transfected cells. N2a cells were transfected with pcDNA3.1-vector or MANF-Flag plasmid. Twenty-four hours after transfection, the cells were treated with Aβ_1–42_ (10 μM) for indicated times and processed for the MTT assay. *P < 0.05, compared to vector-transfected cells at corresponding time points. **c** Effect of endogenous MANF knockdown on the vulnerability to Aβ_1–42_ (10 μM) cytotoxicity in N2a cells. N2a cells were transfected with NC-siRNA or effective MANF-siRNA. Twenty-four hours after transfection, the cells were treated with Aβ_1–42_ (10 μM) for indicated times and processed for the MTT assay. *P < 0.05, compared to NC-siRNA-transfected cells at according time points. **d** Effect of MANF on cell apoptosis induced by Aβ_1–42_. N2a cells were transiently transfected with MANF-Flag or MANF-siRNA followed by treatment of Aβ_1–42_ (10 μM) for another 24 h. TUNEL staining to determine the apoptotic cells. The nuclei were stained with DAPI. Scale bar = 100 μm. **e** Quantitative analysis of the number of TUNEL-positive cells in **d**. The TUNEL-positive cells were counted in five randomly selected fields from four sections of each group. *P < 0.05, **P < 0.01. **f** The protein levels of CHOP and cleaved caspase-3 were determined in Aβ_1–42_-treated N2a cells transfected with MANF-Flag or MANF-siRNA. GAPDH was used as a loading control. All the quantitative data were expressed as mean ± SD from three independent experiments

We found above that Aβ induced MANF expression in neuronal cell line and cultured primary neurons. Therefore, we hypothesize that induction of MANF is protective for neurons while they suffer insults, such as Aβ exposure. To prove this, we evaluated the effects of MANF on neuronal cell viability induced by Aβ_1–42_ following knockdown or overexpression in N2a cells, to decrease or increase MANF expression (Fig. 4a). It was found that MANF overexpression rescued the cells from the toxicity exerted by 10 μM Aβ_1–42_ to a certain extent compared with vector transfected cells (Fig. 4b). On the contrary, knockdown of endogenous MANF with siRNA further enhanced Aβ toxicity compared with NC-siRNA transfected cells (Fig. 4c). TUNEL staining also showed that MANF gene knockdown dramatically increased the number of TUNEL-positive cells under Aβ exposure compared with that in NC-siRNA transfected cells, whereas MANF overexpression has the opposite effect (Fig. 4d, e). Western blotting showed dramatically increased levels of CHOP and cleaved caspase-3 in MANF knockdown cells following Aβ_1–42_ treatment, which further confirmed the protective effect of MANF on neurotoxicity induced by Aβ_1–42_ (Fig. 4f). More interesting, we noticed that MANF knockdown also slightly increased the number of TUNEL-positive cells and CHOP level even in the absence of Aβ_1–42_ compared with the NC-siRNA transfected cells (Fig. 4d–f), suggesting that MANF is indispensable for neuron viability even under physiological condition.

**Fig. 5:**
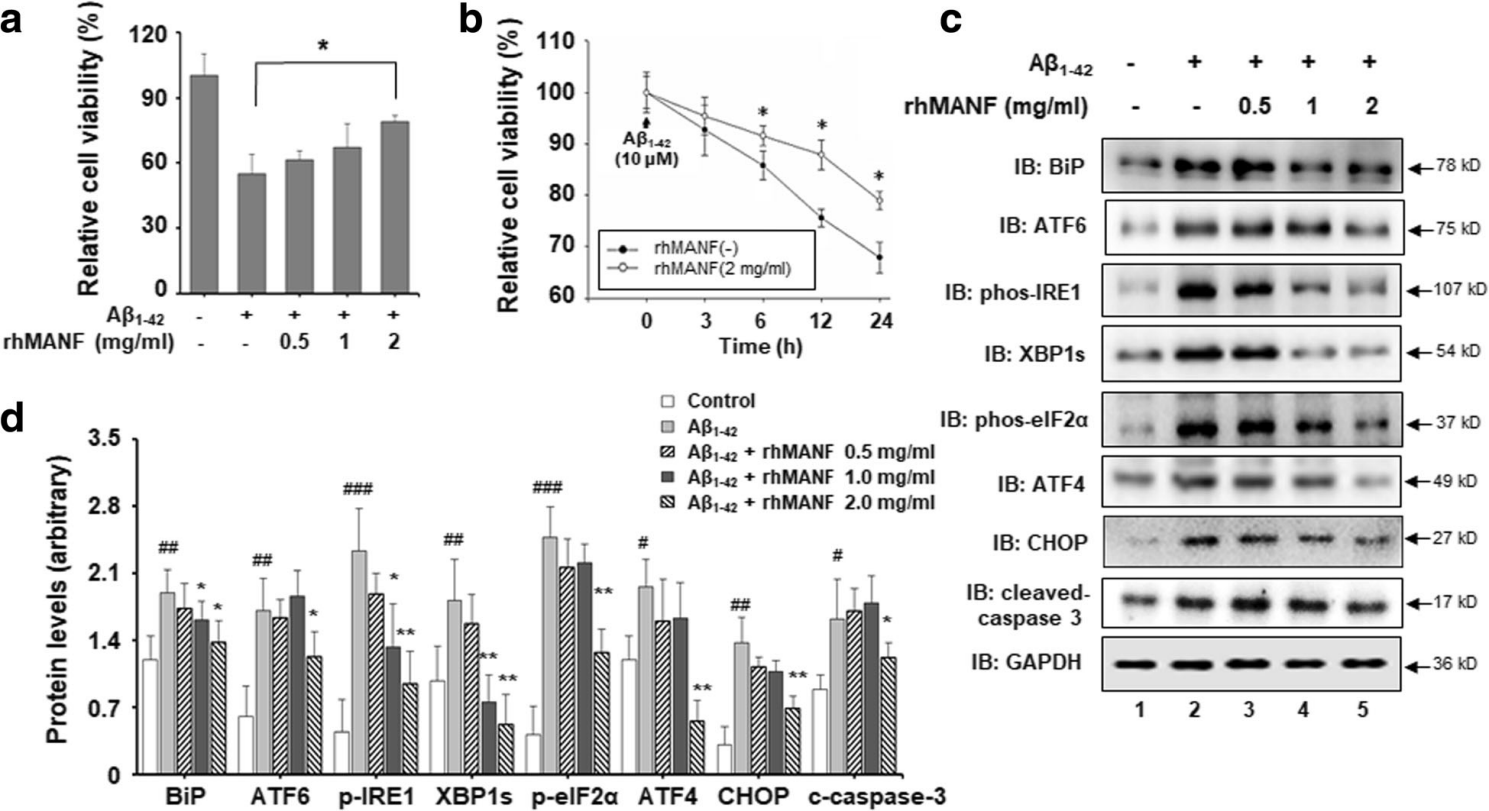
Recombination human MANF protein (rhMANF) protects against Aβ_1–42_-induced cell toxicity. **a** The dose-dependent effect of rhMANF on cell viability in the presence of Aβ_1–42_ exposure. SH-SY5Y cells were pretreated with different concentrations of rhMANF protein (0.5, 1, and 2.0 mg/ml) for 4 h prior to the Aβ_1–42_ (10 μM) treatment for additional 24 h and processed for the MTT assay. *P < 0.05, compared with the cells only treated with Aβ_1–42_. **b** The time-course of rhMANF on cell viability in the presence of Aβ_1–42_. SH-SY5Y were pretreated with rhMANF (2.0 mg/ml) for 4 h following the Aβ_1–42_ (10 μM) treatment for indicated times and processed for the MTT assay. *P < 0.05, compared with the only Aβ_1–42_-treated cells at indicated time points. **c** The protein levels of BiP, ATF6, phospho-IRE1, XBP1s, phospho-eIF2α, ATF4, CHOP, and cleaved caspase-3 were determined in SH-SY5Y cells treated with Aβ_1–42_ (10 μM) for 24 h with or without rhMANF. GAPDH was used as a loading control. **d** The densitometric quantitation of indicated proteins normalized to GAPDH levels in c. *P < 0.05, **P < 0.01, compared with the cells only treated with Aβ_1–42_. ^#^*P* < 0.05, ^##^*P* < 0.01, ^###^*P* < 0.001, compared with control group. All the quantitative data were presented as mean + SD of at least three independent experiments. *C*-*caspase*-*3* cleaved caspase-3

We reported earlier the function of secreting MANF in ischemic brain [28, 38, 39]. To further assess whether secreted MANF plays a neuroprotective role against Aβ toxicity, we evaluated the effect of recombinant human MANF protein (rhMANF) on Aβ_1–42_-induced cell toxicity. Recombinant human MANF was expressed and purified as we previously described [29]. SH-SY5Y cells were pretreated with various concentrations of rhMANF (0.5, 1, and 2 mg/ml) for 4 h prior to Aβ_1–42_ treatment. Twenty-four hours after treatment with Aβ_1–42_ (10 μM), cell viability was determined by using the MTT assay. It was found that rhMANF reversed the decreased cell viability induced by Aβ_1–42_ at the dose of 2 mg/ml (Fig. 5a). As showed in Fig. 5b, rhMANF at the dose of 2 mg/ml significantly increased cell viability at different time points, compared with that in only Aβ_1–42_-treated cells in the absence of rhMANF. The levels of cleaved caspase-3 also decreased with rhMANF treatment (Fig. 5c, d). These results confirmed the protective effect of rhMANF against Aβ cytotoxicity.

### MANF protects against Aβ_1–42_ toxicity via attenuating ER stress

**Fig. 6:**
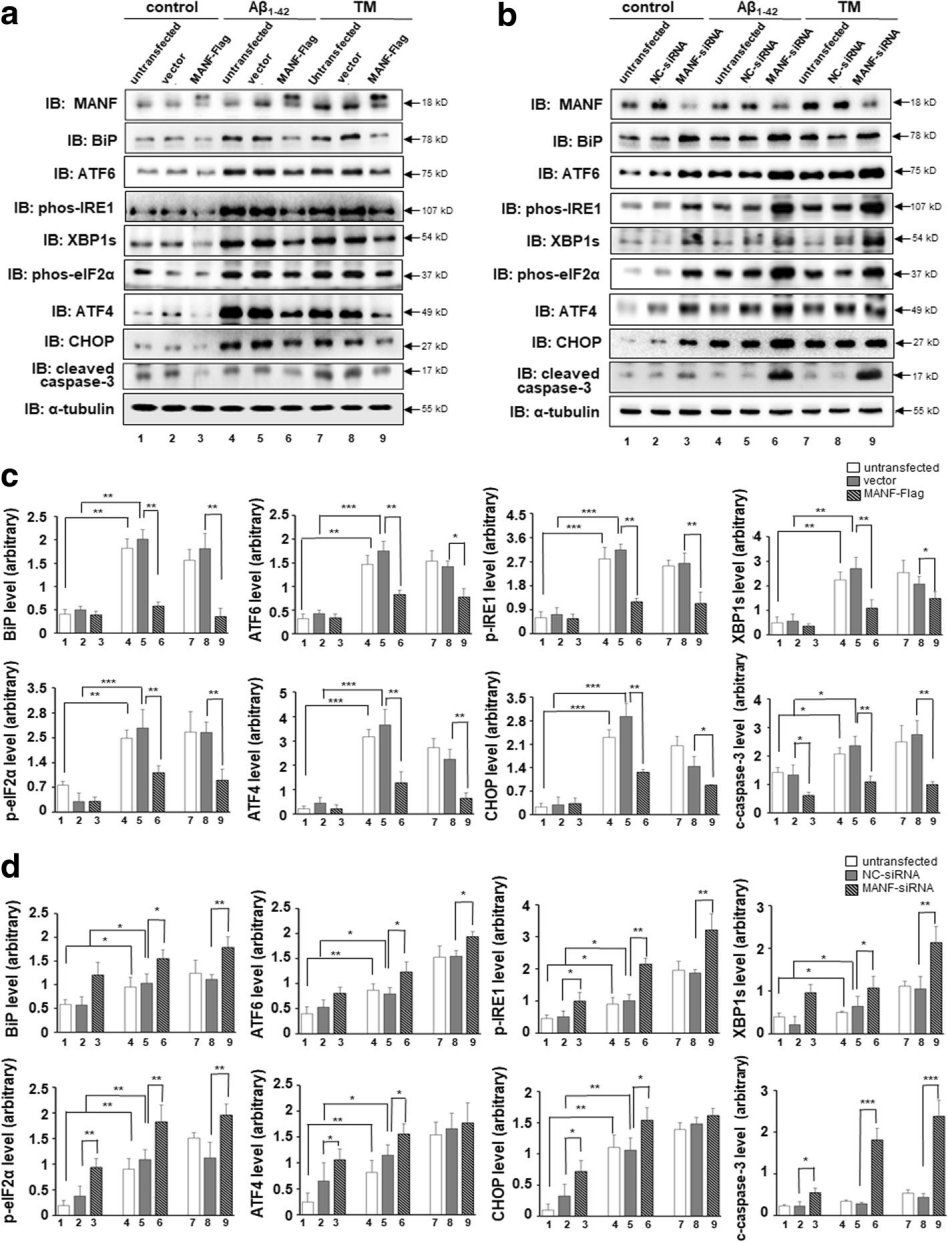
MANF protects against Aβ_1–42_ toxicity via inhibiting ER stress. **a** Effect of MANF overexpression on the levels of BiP, ATF6, phospho-IRE1, XBP1s, phospho-eIF2α, ATF4, CHOP, and cleaved caspase-3 in Aβ_1–42_-treated cells. N2a cells were transfected with pcDNA3.1-MANF-Flag plasmid and its empty vector before Aβ_1–42_ (10 μM) treatment for 24 h or TM (2.5 μg/ml) treatment for 12 h and processed for WB. **b** Effect of MANF knockdown on the levels of BiP, ATF6, phospho-IRE1, XBP1s, phospho-eIF2α, ATF4, CHOP, and cleaved caspase-3 in Aβ_1–42_-treated cells. N2a cells were transfected with NC-siRNA or MANF-siRNA before Aβ_1–42_ (10 μM) treatment for 24 h or TM (2.5 μg/ml) treatment for 12 h, respectively, then processed for WB. **c** Quantitation of proteins normalized to α-tubulin levels in a. **d** Quantitation of proteins normalized to α-tubulin levels in **b**. All the quantitative data were presented as mean ± SD of at least three independent experiments. *P < 0.05, **P < 0.01, ***P < 0.01*. TM* tunicamycin, *C*-*caspase*-*3* cleaved caspase-3

Previous studies have shown that MANF protects against ER stress-induced cell death by regulating UPR-related genes in cerebral ischemia model [28, 29]. We wonder whether MANF attenuated Aβ-induced toxicity by alleviating ER stress. To test this hypothesis, MANF-Flag plasmid or MANF-siRNA was transfected into N2a cells to overexpress or knockdown the endogenous MANF, respectively. We found that MANF overexpression significantly decreased the levels of ER stress-related molecules, such as BiP, ATF6, phospho-IRE1, spliced-XBP1 (XBP1s), phospho-eIF2α, ATF4, and CHOP in Aβ_1–42_-treated cells compared with that in vector transfected cells (Fig. 6a, c, lane 6 vs lane 5). Similarly, the levels of apoptosis marker cleaved caspase-3 also decreased in MANF-Flag transfected cells following by Aβ_1–42_ exposure (Fig. 6a, and c, lane 6 vs lane 5). On the contrary, MANF knockdown upregulated BiP, ATF6, phospho-IRE1, XBP1s, phospho-eIF2α, ATF4, CHOP, and cleaved caspase-3 expression, compared to that of NC-siRNA transfected cells (Fig. 6b, d, lane 6 vs lane 5). We also found that MANF decreased the levels of these UPR-related proteins induced by TM (Fig. 6a–d, lane 9 vs lane 8). These results suggest that MANF alleviates Aβ-induced ER stress via suppressing the UPR signaling pathway activation, which may contribute to neuron protection.

More interesting, MANF knockdown increased BiP level to a certain extent, although no significant difference of BiP expression between MANF-Flag transfected cells and vector transfected cells was observed (Fig. 6a–d, lane 2 vs lane 3). Furthermore, reduced levels of ATF6, phospho-IRE1, phospho-eIF2α, ATF4, and CHOP were also found in MANF knockdown cells in the absence of Aβ_1–42_ or TM (Fig. 6b, lane 2 vs lane 3), suggesting that MANF might relieve ER stress to maintaining cellular homeostasis under physiological conditions.

To clarify whether the rhMANF has the similar effects on the Aβ-induced ER stress, UPR-related proteins were determined by using Western blot in SY5Y cells incubated with rhMANF and Aβ_1–42_. It was found that 2 mg/ml rhMANF decreased the levels of BiP, ATF6, phospho-IRE1, phospho-eIF2α, XBP1s, ATF4, and CHOP in a dose-dependent manner in Aβ_1–42_-treated cells (Fig. 5c, d), suggesting that rhMANF also alleviates Aβ-induced ER stress.

## Discussion

In this study, we demonstrated the roles of MANF in protecting neuronal cells from the toxicity induced by Aβ. We showed here that MANF expression is upregulated in cultured neuronal cells exposure to Aβ_1–42_ as well as in the brains of APP/PS1 mice. Over-expression of MANF or rhMANF treatment protected against Aβ_1–42_-induced cell death in N2a cells and SH-SY5Y cells, whereas MANF gene knockdown aggravated Aβ cytotoxicity. In addition, neuroprotective effect of MANF against Aβ_1–42_-induced cytotoxicity was associated with the inhibition of ER stress, which demonstrated by suppression of UPR signaling cascades and the decrease of CHOP expression and caspase-3 cleavage. These results suggested that MANF may serve as a plausible therapeutic target for the treatment of AD.

It was reported that Aβ induces ER stress leading to neuron apoptosis, and ultimately contributes to the pathogenesis of AD [40–42]. In the present study, we found that Aβ_1–42_ dose- and time-dependently decreased cell viability and increased cell apoptosis. Further investigation showed that the expressions of UPR-related proteins were upregulated both in primarily cultured rat neurons and in SH-SY5Y cells under Aβ exposure, including BiP, ATF6, phospho-IRE1, XBP1s, phospho-eIF2α, ATF4, and CHOP. These results indicate that Aβ_1–42_ induces ER stress, which mediates neuronal cell apoptosis. Although molecular mechanisms of ER stress-mediated Aβ neurotoxicity still remain obscure, many studies have confirmed that Aβ triggers the UPR in neurons, which accompanies the activation of protective pathways such as BiP and PERK-eIF2a pathway, as well as the apoptotic pathways of the UPR such as CHOP and caspase-4 [43].

Our previous studies had identified MANF as 1 of the 12 commonly UPR upregulated genes, and ER stress inducer TM induced MANF expression in the primarily cultured neurons [13]. The mammalian MANF promoter contains ER stress-responsive elements ERSE and ERSEII, which are important for XBP1s and ATF6 binding leading to upregulation of MANF expression in response to ER stress [14, 22, 44, 45]. In this study, Aβ_1–42_ treatment significantly increased the expressions of XBP1s and ATF6, which at least partially contributes to the upregulation of MANF accompanied by subsequently increased expression of other UPR related proteins. Consistent with our results in cultured neuronal cells with Aβ, the expression of MANF was also dramatically upregulated in APP/PS1 mice compared with those of age- and sex-matched wild-type mice, especially in 6-month-old APP/PS1 mice, suggested that increased MANF expression is correlated with the severity of AD. But there is no difference of MANF level was observed between 4-month and 6-month-old wild-type mice, which suggested that aging is not likely to be responsible for the upregulation of MANF. Meanwhile, we found that MANF is mostly expressed in neurons in the brains of APP/PS1 mice, which is in accordance with the characteristics of MANF expression in rat ischemia model [27, 29]. Though some NeuN-negative cells also presented MANF immunoreactivity, the function of MANF in these cells needs further study. In addition, we noticed that the immunoreactivity of MANF, BiP, and CHOP is weak and diffused in the cytoplasm of SY5Y cells without Aβ_1–42_, whereas increased perinuclear MANF immune-positive signals were observed in most of the cell treated with Aβ_1–42_ or TM as well as in the APP/PS1 mice brain. Meanwhile, we found the significant upregulated immunoreactivity of BiP in cytoplasm and nuclear translocation of CHOP in the cells treated with Aβ_1–42_ or TM, which implies UPR activation. The cellular distribution of MANF under different pathology may be an interesting question. Our previous studies showed that MANF localization primarily in the juxta-nuclear region in the TM-treated cells, which proved to be localized in the ER and Golgi [13]. Conversely, MANF induced by the stimulation of cerebral ischemia had no overlap with DAPI, suggesting that MANF localizes in cytosol and focal ischemia did not induce its relocalization [29], whereas nuclear MANF immune-positive signals were observed in a few TM or LPS-treated primarily cultured fibroblast-like synoviocytes as well as in the synovial tissue of antigen-induced arthritis rabbit model [23]. The reason for MANF exhibits different cellular distribution under different pathology worthy of further investigation.

MANF is a new member of neurotrophic factor families, which has been shown to be upregulated during ER stress and protected several cell populations from ER stress-induced cell death in vivo or in vitro [13, 19, 31, 46], especially in PD and cerebral ischemia [19, 47, 48]. In this study, we showed that over-expression of MANF attenuated Aβ_1–42_-induced neuronal cell death, whereas MANF gene knockdown aggravated the Aβ_1–42_ neurotoxicity. Whether MANF is directly involved in the regulation of UPR and thus play a key role in cell protection is still obscure. Previous studies reported that MANF may facilitate the formation of cysteine bridges and protein folding in the ER, thus reducing the ER stress caused by accumulation of unfolded or misfolded proteins [49]. The most convenient and direct evidence is pancreas-specific Manf^−/−^ mice developed severe insulin-dependent diabetes due to progressive reduction of β cell mass and activation of UPR, including spliced Xbp1 and Chop mRNA, which implicates that MANF deficiency may lead to activation of the UPR following unresolved ER stress [32]. Our previous studies also suggested that intraventricular injection of rhMANF reduced the elevated levels of BiP/Grp78, phosp-IRE1, and XBP1s induced by focal cerebral ischemia, but not affect CHOP expression [28]. Glial cell line-derived neurotrophic factor (GDNF), another neurotrophic factor, was proved to prevent the translocation of CHOP into the nucleus in the brains of rabbits by intracisternal injections of Aβ [50]. In this study, we observed that the levels of UPR-related molecules BiP, ATF6, phospho-IRE1, XBP1s, phospho-eIF2α, ATF4, CHOP, and apoptosis marker cleaved caspase-3 reduced in different degree in MANF over-expressed cells compared to vector-transfected cells, whereas MANF knockdown further increased these UPR relative proteins, suggesting that MANF alleviate Aβ-induced ER stress via suppressing UPR signaling activation, which lead to a decrease of CHOP and caspase-3 cleavage. In addition, our current data showed that all of the three UPR sensors were activated by Aβ in some extent, since the fact that CHOP is a transcriptional target of not only ATF4 but also XBP-1 and ATF6 provides an obvious possible link among all three branches [51, 52], which pathway in Aβ pathology is the leading cause for CHOP induction and neuron death needs to further exploration.

On the other hand, the crystal structure analysis revealed that the C-terminal domain of MANF (C-MANF) shares the highest structural homology with the SAP domain of Ku70 protein, a well-known inhibitor of pro-apoptotic Bcl-2-associated X protein (Bax) [53]. This was confirmed by the cellular studies that MANF protected neurons as efficiently as Ku70 did [53, 54]. Based on this, we cannot exclude the possibility that the direct anti-apoptosis activity of MANF partially contributes to its protective effect against Aβ neurotoxicity.

Most importantly, MANF was reported to act as both a secreted neurotrophic factor and an intracellular protein [55]. Previous studies have suggested a secretion-based protective role of MANF in cerebral ischemia and myocardial ischemia [27–29]. Conversely, no protected effects against the Bax-dependent apoptosis were observed when rhMANF was added to the culture medium of cultured newborn mouse SCG neurons directly the neurons [53]. Although rhMANF has been commonly used for the study regarding the function of secretory MANF protein, different concentrations of rhMANF was adopted in vivo and in vitro experiments depend on the different types of cells or the disease models [23, 24, 26, 28–31, 38, 39, 48, 56–58]. Here, we tried several concentrations of rhMANF in preliminary experiments and finally we found that the concentrations of 0.5, 1, and 2 mg/ml rhMANF are sufficient to protect against the Aβ-induced neurotoxicity and alleviate ER stress. To date, signal transducing receptor for MANF has not been identified, although protein kinase C (PKC) signaling has been described to be activated downstream of MANF [58]. Therefore, whether and how MANF acts in autocrine/paracrine manner will need more studies.

## Conclusions

MANF protects against Aβ toxicity by attenuating Aβ-induced ER stress, which may be one of the potential targets for the neurodegenerative diseases related to pathological ER stress, including AD.

## Additional files

Additional file 1: **Figure S1.** Aβ_1–42_ induces ER stress and upregulates MANF expression in primarily cultured rat neurons. Primarily cultured neurons at day 10–14 were treated with serum-free DMEM, DMEM containing Aβ_25–35_ (10 μM) for 24 h, or DMEM containing TM (2.5 μg/ml) for 12 h, respectively. (a) Representative images of MANF (green) and BiP (red) immunofluorescence labeling of neurons. (b) Representative images of MANF (green) and CHOP (red) immunofluorescence labeling of neurons. The nuclei were stained with DAPI (blue). Scale bar = 40 μm. TM: tunicamycin. (TIF 2057 kb)

Additional file 2: **Figure S2.** Representative images of Aβ deposition in the brains of APP/PS1 transgenic mice. Immunohistochemical staining showed obvious Aβ aggregation in cortex (a) and hippocampus (c) of 6-month-old APP/PS1 mice. Panels b and d are enlarged images of framed rectangle in a and c, respectively. Scale bar = 20 μm. (TIF 5129 kb)

Additional file 3: **Figure S3.** The expressions of BiP and CHOP in the brains of the APP/PS1 transgenic mice and age- and sex-matched WT mice, respectively. (a) Immunofluorescence labeling of BiP (green) in hippocampus and cortex of WT mice (upper panel) and APP/PS1 mice aged 6 months (lower panel). (**b**) Immunofluorescence labeling of CHOP (green) in hippocampus and cortex of WT mice (upper panel) and APP/PS1 mice aged 6 months (lower panel). The nuclei were stained with DAPI (blue). Scale bar = 100 μm (TIF 6442 kb)

## Abbreviations

AD: Alzheimer’s disease
APP: Amyloid precursor protein
ATF6: Activating transcription factor 6
Aβ: Amyloid β-peptide
Bax: Bcl-2-associated X protein
BiP: Immunoglobulin-binding protein
CDNF: Cerebral dopamine neurotrophic factor
CHOP: C/EBP homologous protein
DAPI: 4′,6-Diamidino-2-phenylindole
DMEM: Dulbecco’s modified Eagle’s medium
EIF2a: Eukaryotic translation initiation factor 2A
ER: Endoplasmic reticulum
IRE-1: Inositol requiring kinase 1
MANF: Mesencephalic astrocyte-derived neurotrophic factor
MTT: Methyl thiazolyl tetrazolium
NTFs: Neurotrophic factors
PBS: Phosphate-buffered saline
PERK: PKR-like ER kinase
TM: Tunicamycin
UPR: Unfolded protein response
