# Supplementary figures and images for: Mesencephalic astrocyte-derived neurotrophic factor (MANF) protects against Aβ toxicity via attenuating Aβ-induced endoplasmic reticulum stress
Source: J Neuroinflammation. 2019 Feb 13;16:35. doi: 10.1186/s12974-019-1429-0 (PMC6373169; doi:10.1186/s12974-019-1429-0)

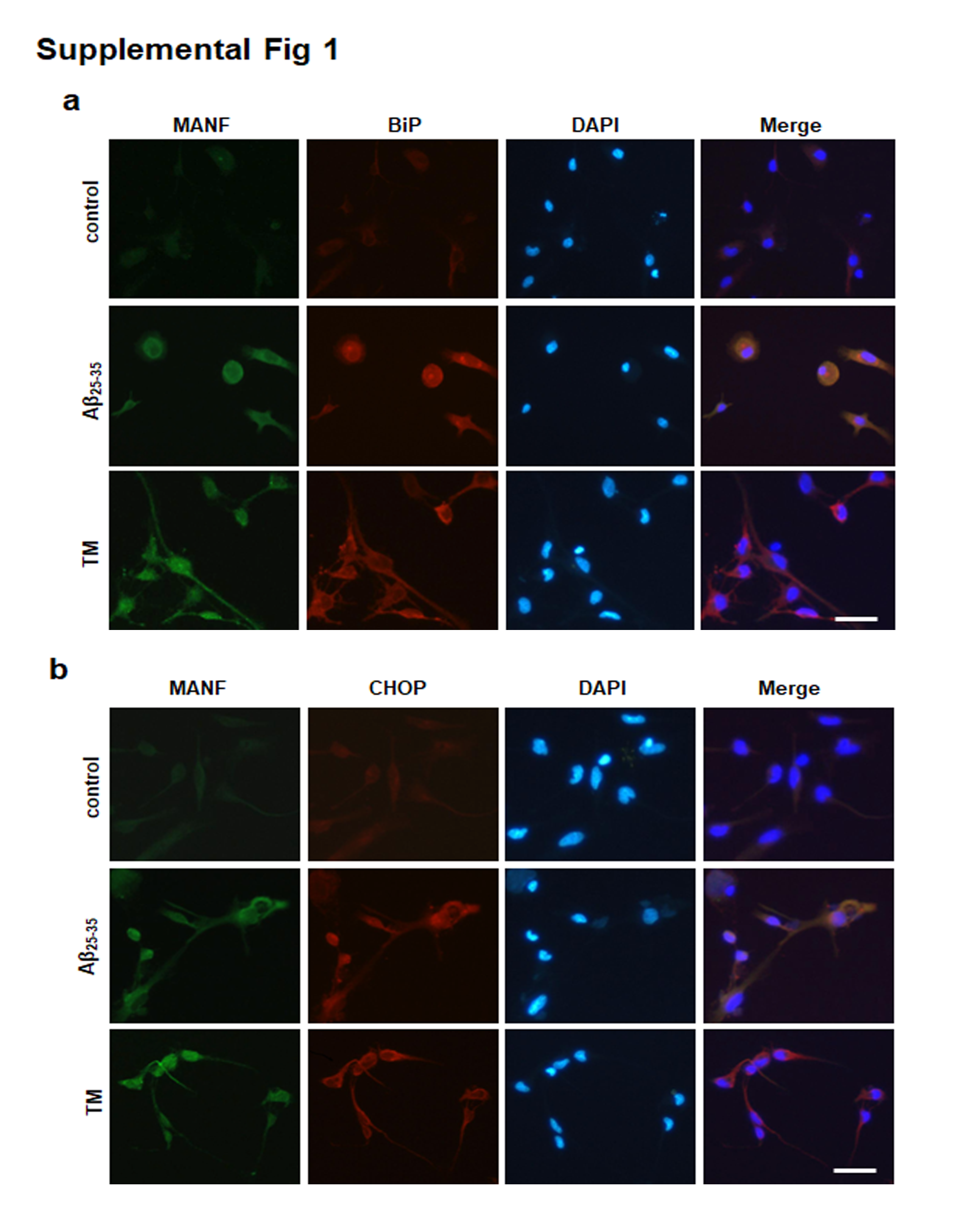

Supplement: Supplementary file 1 — Figure S1. Aβ1–42 induces ER stress and upregulates MANF expression in primarily cultured rat neurons. Primarily cultured neurons at day 10–14 were treated with serum-free DMEM, DMEM containing Aβ25–35 (10 μM) for 24 h, or DMEM containing TM (2.5 μg/ml) for 12 h, respectively. (a) Representative images of MANF (green) and BiP (red) immunofluorescence labeling of neurons. (b) Representative images of MANF (green) and CHOP (red) immunofluorescence labeling of neurons. The nuclei were stained with DAPI (blue). Scale bar = 40 μm. TM: tunicamycin. (TIF 2057 kb) [file 12974_2019_1429_MOESM1_ESM.tif]

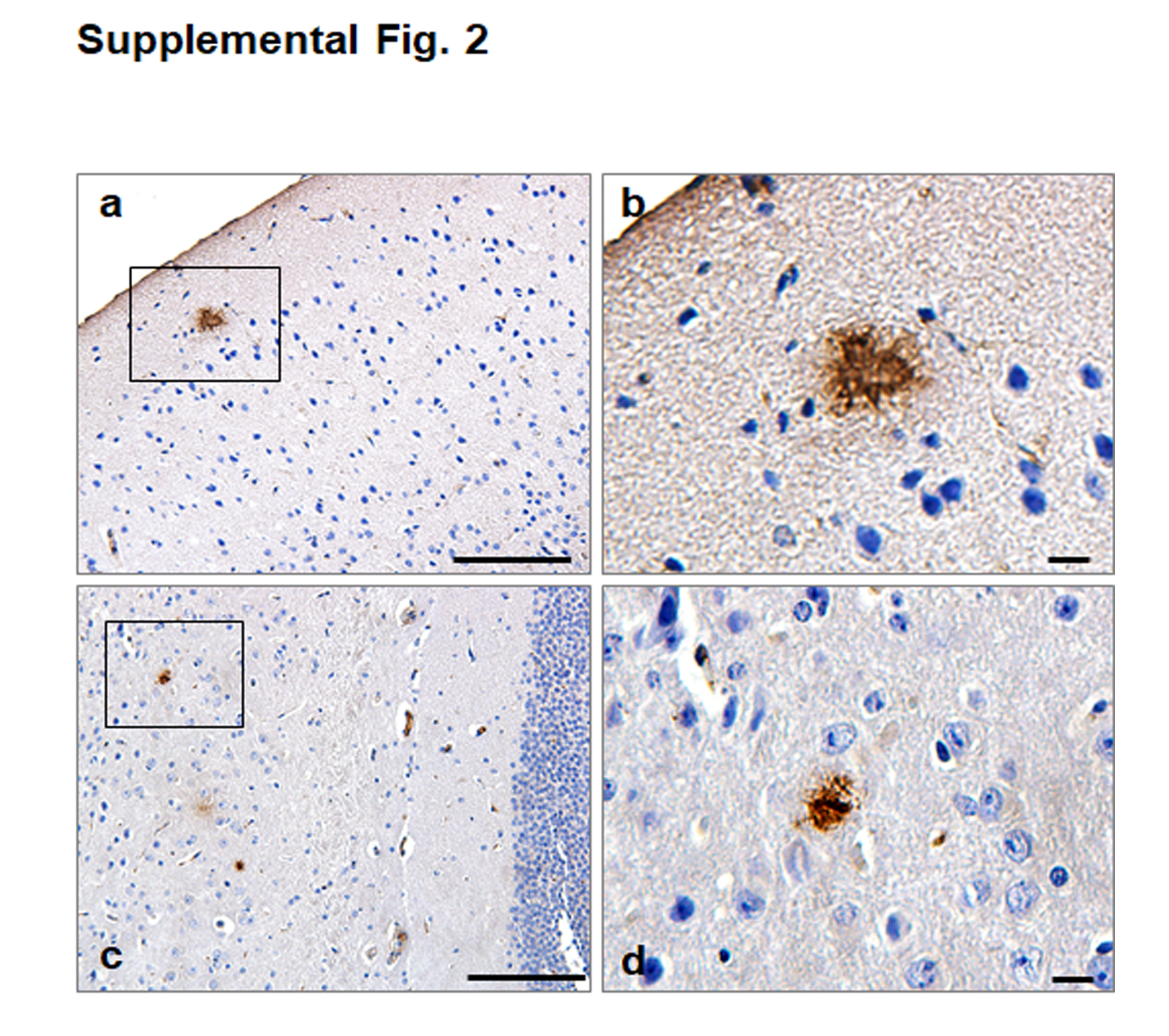

Supplement: Supplementary file 2 — Figure S2. Representative images of Aβ deposition in the brains of APP/PS1 transgenic mice. Immunohistochemical staining showed obvious Aβ aggregation in cortex (a) and hippocampus (c) of 6-month-old APP/PS1 mice. Panels b and d are enlarged images of framed rectangle in a and c, respectively. Scale bar = 20 μm. (TIF 5129 kb) [file 12974_2019_1429_MOESM2_ESM.tif]

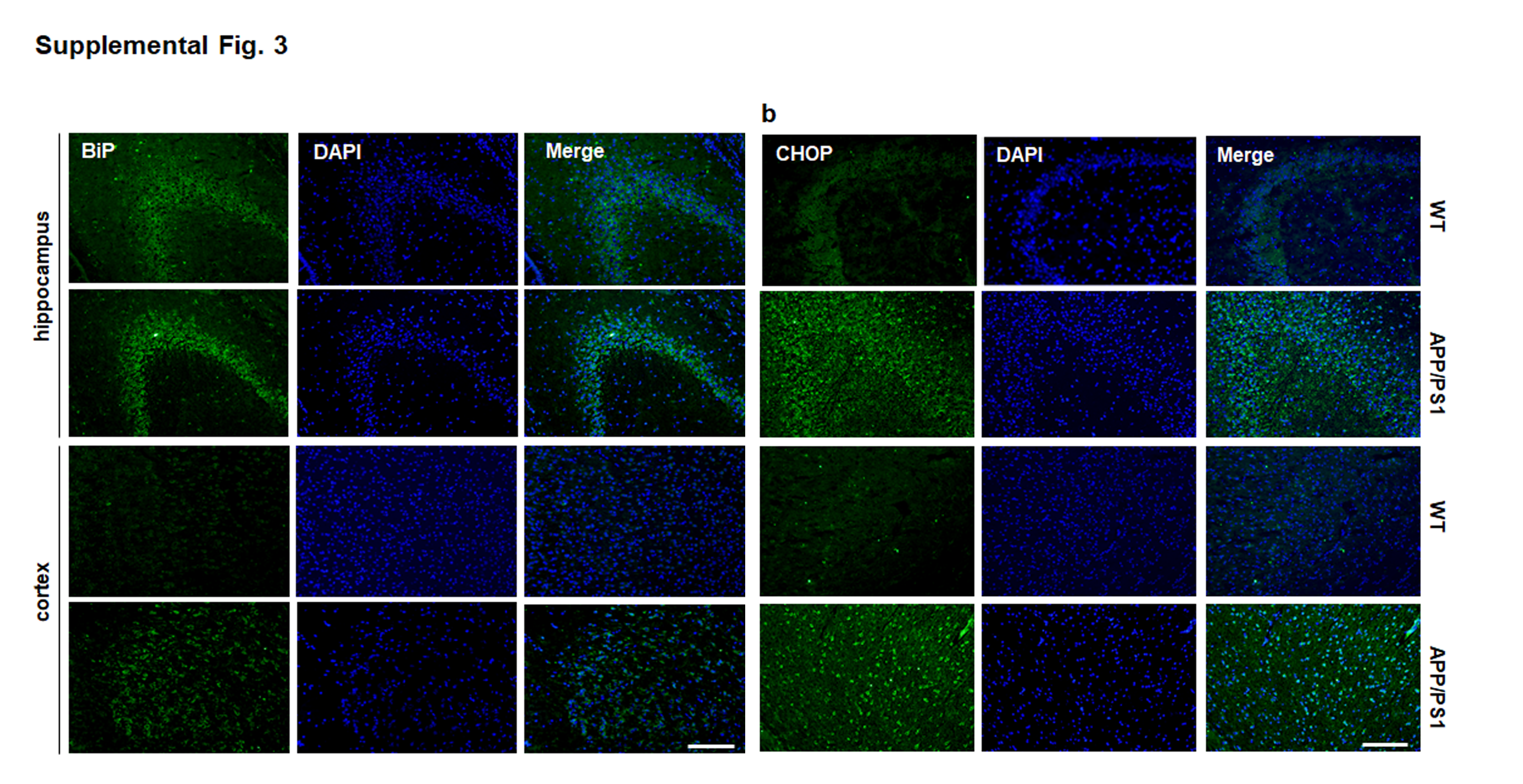

Supplement: Supplementary file 3 — Figure S3. The expressions of BiP and CHOP in the brains of the APP/PS1 transgenic mice and age- and sex-matched WT mice, respectively. (a) Immunofluorescence labeling of BiP (green) in hippocampus and cortex of WT mice (upper panel) and APP/PS1 mice aged 6 months (lower panel). (b) Immunofluorescence labeling of CHOP (green) in hippocampus and cortex of WT mice (upper panel) and APP/PS1 mice aged 6 months (lower panel). The nuclei were stained with DAPI (blue). Scale bar = 100 μm (TIF 6442 kb) [file 12974_2019_1429_MOESM3_ESM.tif]
